# Legislation and Current Practices Concerning Risk Assessment of Skin Sensitizers in the European Union: A Comparative and Survey Study

**DOI:** 10.1111/cod.14754

**Published:** 2025-02-07

**Authors:** Mathias Krogh Pedersen, Jakob Ferløv Baselius Schwensen, Jose Hernán Alfonso, Steen Mollerup, Gianluca Selvestrel, Christina Rudén, Martin F. Wilks, Jeanne Duus Johansen

**Affiliations:** ^1^ The National Allergy Research Centre (Videncenter for Allergi), Department of Allergy, Dermatology and Venerology Copenhagen University Hospital Herlev‐Gentofte, Gentofte Hospital Hellerup Denmark; ^2^ Faculty of Health and Medical Sciences, Department of Clinical Medicine University of Copenhagen Copenhagen Denmark; ^3^ Section of Occupational Medicine and Epidemiology National Institute of Occupational Health Oslo Norway; ^4^ Department of Dermatology Oslo University Hospital, Rikshospitalet Oslo Norway; ^5^ Section of Occupational Toxicology National Institute of Occupational Health Oslo Norway; ^6^ Laboratory of Environmental Chemistry and Toxicology, Environmental Health Department Istituto di Ricerche Farmacologiche Mario Negri IRCCS Milano Italy; ^7^ Division of Toxicology Wageningen University & Research Wageningen Netherlands; ^8^ Department of Environmental Science Stockholm University Stockholm Sweden; ^9^ Swiss Centre for Applied Human Toxicology & Department of Pharmaceutical Sciences University of Basel Basel Switzerland

**Keywords:** allergic contact dermatitis, contact allergy, regulatory status, risk assessment, skin sensitization

## Abstract

**Background:**

Despite legislation aimed to protect the population against skin sensitization in the European Union (EU), over one quarter of the general population is sensitised to at least one chemical.

**Objectives:**

To provide an overview and comparison of European legislation concerning skin sensitization. In addition, we gathered the opinions of experts and stakeholders regarding improvements in the legislation and risk assessment process in the EU, to provide suggestions for improvement.

**Methods:**

Legislation was identified and compared. Four questionnaires were created towards industry, competent authorities and regulators, researchers/clinicians, and non‐governmental organisations. The questions concerned the legislation, the risk assessment process, data collection and potential improvements.

**Results:**

Seven areas of legislation were analysed. The legislation was found to be unharmonised, for example, differing modes of restriction and accepted tests for skin sensitization. Approximately 40% of the questionnaire respondents found that the EU legislation and tools were not sufficiently protective. To improve the legislation 83% suggested harmonisation and 68% suggested better data sharing. Other areas were: improved exposure data (78%), better understanding of the skin sensitization mechanism (67%) and non‐animal tests (66%).

**Conclusions:**

Stakeholders had varying confidence towards the protection of European citizens against skin sensitization. Multiple areas for improvement regarding the legislations and the risk assessment process were identified.

## Graphical abstract

Through an analysis of EU legislation and questionaries distributed to expert and stakeholders, this paper investigates the legislation and risk assessment process in EU concerning skin sensitizers. Approximately 40% of the participants found the current protection insufficient and multiple areas of improvement were identified and discussed.

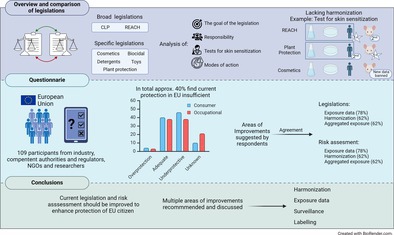

## 1 Introduction

The European Union (EU) aims to protect against skin sensitization caused by exposure to sensitising chemicals through legislation. The legislation lays down rules for, for example, test requirements, risk assessment procedures, mandatory ingredient labelling and upper concentration limits of sensitising chemicals.

The two broadest aeas of legislation targeting chemicals in the EU include the regulation on Classification, Labelling and Packaging (EC) No. 1272/2008, (CLP) and the regulation on Registration, Evaluation, Authorization and Restriction of Chemicals (EC) No. 1907/2006 (REACH). Other legislation concerns substances/products for specific usages such as cosmetics, toys, plant protection, detergents and biocides. The legislation has been launched at different points in time in the process of creating the internal market in the EU. They have also to a large extent been managed independently of each other with different agencies and authorities involved in their implementation. The European Chemicals Agency (ECHA) has multiple roles in the implementation of the CLP, REACH and the Biocidal Products Regulation (BPR) including guidance and creation of inventories [1, 2, 3]. The European Food Safety Authority (EFSA) is involved in implementing the Plant Protection Products Regulation (PPP) giving scientific advice and risk assessments [4]. The Scientific Committee of Consumer Safety (SCCS) provides opinions and guidance regarding cosmetic products [5]. Very few, if any, attempts have so far been made to harmonise these regulations, despite their (potential) importance for health and safety.

The success of legislation targeting health effects would usually be measured against their effectiveness in reducing the problem in focus. Skin sensitization continues to impact a significant number of individuals across various groups, including the general adult population [6], adolescents [7] individuals in specific occupations [8], and those with eczema [9]. In total 27% of the general population in the EU are sensitised to at least one chemical [6] (i.e., ~100 million citizens). Skin sensitization is a pre‐state of allergic contact dermatitis (ACD), which develops in a sensitised individual depending on exposure to the ascertained allergen, and a substantial part of sensitised individuals will have, or have had, ACD [6]. The most common skin sensitizers are fragrances, preservatives, and metals [6] ACD is a chronic disease associated with a considerable burden in terms of medical care, decreased work ability and quality of life. It has been shown that young people with occupational contact dermatitis are especially affected [10]. The expenses for society are considerable and in the range of 22 to 32 billion €/year based on recent socio‐economic estimations for skin sensitization to fragrances alone [11]. Hence, they are likely to be an underestimation of the true total cost. For occupational contact dermatitis, the related costs exceed 5 billion €/year in the EU by loss of productivity, sick‐leave and job loss [12]. As skin sensitization/ACD is an environmental and public health problem, the preventive potential at the population level is substantial.

This survey investigation aims to: (1) analyse and compare the major regulations/directives concerning the management of skin sensitizers, (2) examine the current practices of risk assessment for skin sensitizers conducted by different stakeholders and (3) identify scientifically unfounded differences, overlaps and potential areas of improvement.

This work is a part of the European Partnership for the Assessment of Risks from Chemicals (PARC) project financed under the Horizon Europe framework programme for 2022–2028 (https://www.eu‐parc.eu). PARC is a major chemical risk assessment program that was established to develop and implement Next Generation Risk Assessment (NGRA) to protect human health and the environment [13].

## 2 Materials and Methods

### 2.1 Comparison of Legislation

The EU legislation regulating the use of chemicals intended for skin contact, or where accidental skin contact may happen, were identified, and compared. The following legislation was included: The CLP regulation (EC) No. 1272/2008 [2], the REACH Regulation (EC) No. 1907/2006 [1], the Cosmetic Products Regulation (EC) No. 1223/2009 [5], the Detergents Regulation (EC) No. 648/2004 [14], the Biocidal Products Regulation (EU) No. 528/2012 [3], the Toys directive 2009/48/EC [15] and the Plant Protection Products Regulation (EC) No. 1107/2009 [4] (including data requirement regulation (EU) No. 283/2013 [16]). The latest consolidated version of the legislation (at the time of first reading: 30/01/2023) was found on EUR‐Lex [17]. The regulations and directives were systematically scrutinised, and all information deemed relevant for skin sensitization was collected. The legislation was analysed and compared across four areas: (1) the goal of the legislation, (2) who is legally responsible (responsibility), (3) which methods can be used to investigate the skin sensitising properties (in vivo and alternative non‐animal tests) and (4) how the legislation acts to protect against skin sensitization (mode of action).

### 2.2 Questionnaire

Four questionnaires (Data [Link], [Link]) were created for the four different target groups: (1) Industry, (2) competent authorities and regulators, (3) researchers/clinicians and (4) Non‐Governmental Organisations (NGOs). The answers to the questionnaires were collected and managed using REDCap electronic data capture tools hosted at the capital region of Denmark. All questions were developed for the purpose of this study, and a pre‐evaluation was performed by 12 relevant test persons from three of the groups: (1) competent authorities and regulators, (2) researchers/clinicians and (3) NGOs. The test persons filled in the questionnaires and were subsequently interviewed to ensure that the questions were relevant and easy to understand.

All questionnaires were sent out to the participants by email in the period from 2023‐May‐09 to 2023‐Aug‐28 (the study population and response frequency are described and discussed in Section 3.2). For all groups, except researchers/clinicians, two reminders were sent 2 weeks apart. Researchers/clinicians were contacted through the European Society of Contact Dermatitis. This group was only contacted once for technical reasons. In total, the questionnaires were opened 283 times and 109 unique answers were given. Initially, 125 industry, 107 competent authorities and regulators, 72 NGOs and all members of the European Society of Contact Dermatitis were contacted. This reach‐out resulted in 44 answers from regulators and competent authorities, 31 from researchers, 24 from industry and 10 from NGOs.

The questionnaires concerned their opinions on the contents and effects of the current legislation and risk assessment methodologies, including which risk assessment methods are used, the type of data collected, areas of improvement and their assessments of the current protection levels for consumer/occupational products at a rating from 1 to 5 (worst‐best). The participants in all groups, except researchers/clinicians, were found through EU wide umbrella organisations (such as the European Chemicals Agency (ECHA), The European Consumer Organisation (BEUC) and The European Chemical Industry Council (Cefic)). Many questions contained an option to add a free text comment. All participants were provided with enough information to give informed consent when entering the study and relevant data security steps were taken. No sensitive personal data was collected, and the identity of individuals/organisations cannot be disclosed.

No ethical approval is needed for this kind of study in Denmark. Data approval was obtained.

### 2.3 Statistics

Descriptive statistics (percentages) were calculated using IBM SPSS statistics 28 64‐bit and Microsoft Excel for Microsoft 365 MSO (Version 2208 Build 16.0.15601.20796) 32‐bit.

## 3 Results

### 3.1 Comparison of Legislation

A collection of legislation in the EU related to skin sensitization was assembled, and details from the comparisons of the four areas are shown in Table 1A–D.

**TABLE 1:** (A–D) Legislative texts assembled and used for comparison.

(A) The goal of the legislation	
CLP [2]	“The purpose of this Regulation is to ensure a high level of protection of human health and the environment as well as the free movement of substances, mixtures and articles” (title I, art. 1)
REACH [1]	“The purpose of this Regulation is to ensure a high level of protection of human health and the environment, including the promotion of alternative methods for assessment of hazards of substances, as well as the free circulation of substances on the internal market while enhancing competitiveness and innovation” (title 1, art. 1)
Cosmetics Regulation [5]	“This Regulation establishes rules to be complied with by any cosmetic product made available on the market, in order to ensure the functioning of the internal market and a high level of protection of human health” (chap. I, art. 1)
Biocidal Products Regulation [3]	“The purpose of this Regulation is to improve the functioning of the internal market through the harmonisation of the rules on the making available on the market and the use of biocidal products, whilst ensuring a high level of protection of both human and animal health and the environment. The provisions of this Regulation are underpinned by the precautionary principle, the aim of which is to safeguard the health of humans, the health of animals and the environment. Particular attention shall be paid to the protection of vulnerable groups” (chap. I, art. 1)
Toys Directive [15]	“This Directive lays down rules on the safety of toys and on their free movement in the Community” (chap. 1, art. 1)
Detergents Regulation [16]	“This Regulation establishes rules designed to achieve the free movement of detergents and surfactants for detergents in the internal market while, at the same time, ensuring a high degree of protection of the environment and human health” (art. 1)
Plant Protection Products [4]	“The purpose of this Regulation is to ensure a high level of protection of both human and animal health and the environment and to improve the functioning of the internal market through the harmonisation of the rules on the placing on the market of plant protection products, while improving agricultural production” (chap. 1, art. 1)

(B) Responsibility	
CLP [2]	Manufacturers, importers and downstream users of a substance shall identify the relevant available information for the purposes of determining whether the substance entails a physical, health or environmental hazard as set out in annex I (title II, art. 5)
REACH [1]	Unless otherwise stated this regulation, “any manufacturer or importer of a substance, either on its own or in one or more mixture, in quantities of one tonne or more per year shall submit a registration to the Agency” (title II, chap. 1, art. 6)
Cosmetics Regulation [5]	“Only cosmetic products for which a legal or natural person is designated within the Community as ‘responsible person’ shall be placed on the market” (chap. II, art. 4)
Biocidal Products Regulation [3]	Active substances have a longer chain of responsible parties before being placed on a market. The applicant must submit a summary of the product to ECHA. A member state authority must be chosen to evaluate the application. Then the product can be authorised on both a national level or a union level (chap. II, art. 7)
Toys Directive [15]	“Manufacturers shall, before placing a toy on the market, carry out an analysis of the chemical, physical, mechanical, electrical, flammability, hygiene and radioactivity hazards that the toy may present, as well as an assessment of the potential exposure to such hazards” (chap. IV, art. 18)
Detergents Regulation [14]	“Manufacturers shall be responsible for the conformity of detergents and/or of surfactants for detergents with the provisions of this regulation and its annexes” (art. 3.3)
Plant Protection Products [4]	An application for the approval of an active substance or for an amendment to the conditions of an approval shall be submitted by the producer of the active substance. A joint application may be submitted by an association of producers designated by the producers for the purpose of compliance with this Regulation

(C) Test for skin sensitization	
CLP [2]	A wide range of tests can be used to categorise skin sensitizers. Human data: Human Repeat Insult Patch Test (HRIPT), diagnostic patch test data and epidemiological evidence. Animal data: local lymph node assay (LLNA), Guinea Pig Maximisation test (GMPT) and the Buehler guinea pig test If these tests are not sufficient non‐standard methods can be used (annex I, 3.4.2.2.1.3–3.4.2.2.4.3)
REACH [1]	“Before new tests are carried out to determine the properties listed, all available in vitro data, in vivo data, historical human data, data from valid (Q)SARs and data from structurally related substances (read‐across approach) shall be assessed first” (annex VII (c)) If this is information is not sufficient the following test can be used: “In vitro/in chemico tests addressing the following key events: (a) molecular interaction with skin proteins; (b) inflammatory response in keratinocytes; (c) activation of dendritic cells” (annex VII, 8.3.1). “New in vivo testing shall only be performed if the above‐mentioned data are insufficient. The murine local lymph node assay (LLNA) is the first‐choice method for in vivo testing. Only in exceptional circumstances should another test be used. Justification for the use of another in vivo test shall be provided” (annex VII, 8.3.2)
Cosmetics Regulation [5]	“The following shall be prohibited: … The placing on the market of cosmetic products containing ingredients or combinations of ingredients which, in order to meet the requirements of this Regulation, have been the subject of animal testing using a method other than an alternative method (non‐animal methods). Animal tests performed before the ban of animal testing are still accepted” (chap. V, art. 18) “The toxicological profile of substance contained in the cosmetic product for all relevant toxicological endpoints. A particular focus on local toxicity evaluation (skin and eye irritation), skin sensitisation, and in the case of UV absorption photo‐induced toxicity shall be made”
Biocidal Products Regulation [3]	“The assessment shall comprise the following tiers: Assessment of the available human, animal and non‐animal data; Skin sensitisation, in vitro testing… addressing each of the following key events of skin sensitisation: Molecular interaction with skin proteins; Inflammatory response in keratinocytes; Activation of dendritic cells; Skin sensitisation in vivo testing. The Murine Local Lymph Node Assay (LLNA) is the first‐choice method for in vivo testing. Another skin sensitisation test may only be used in exceptional cases. If another skin sensitisation test is used, justification shall be provided” (annex II, title I, item 8.3) New in vivo testing is only allowed if in vitro and in chemico results are not adequate
Toys Directive	N/A
Detergents Regulation	N/A
Plant Protection Products [16]	“The local lymph node assay (LLNA) shall be used, including where appropriate the reduced variant of the assay. In case the LLNA cannot be conducted, a justification shall be provided and the Guinea Pig Maximisation Test shall be performed. Where a guinea pig assay (Maximisation or Buehler), meeting OECD guidelines and providing a clear result, is available, further testing shall not be carried out for animal welfare reasons. The need to perform supplementary studies on the plant protection product shall be discussed with the national competent authorities on a case‐by‐case basis” (annex, part A, sec. 5, 5.2.6) Reports from human studies, when must be considered if they indicate a higher sensitivity than animal experiments (annex, part A, sec. 5, 5.9.2)

(D) Modes of action	
CLP [2]	Label elements shall be used for substances or mixtures meeting the criteria for classification in this hazard class in accordance with tab. 3.4.7. Skin sensitizers are divided into category 1, and subcategory 1A or 1B (skin sensitizer, strong sensitizer and weak sensitizer) (annex I, tab. 3.4.7) The categorisation is based upon specific cut‐off values from in vivo animal methods, and evidence from human sensitization in a weight‐of‐evidence approach. If this is found insufficient, other kinds of data can be used on a case‐by‐case basis (annex I, 3.4.2.2.1.3–3.4.2.2.4.3) Substances or mixtures containing a skin sensitizer at the concentration of 1% (for category 1 and 1B substances) or 0.1% (for category 1A substances) are required to be labelled with corresponding warning GHS pictogram, the signal word “Warning” and hazard statement “H317: May cause an allergic skin reaction” and the precautionary statement prevention: P261, P272 and P280 (annex I, tabs. 3.4.6 and 3.4.7)
REACH [1]	Substances, mixtures or groups of substances can be placed in annex XVII if the risk for skin sensitization is high enough (title VIII, chap. 1, art. 67) (as in the case of nickel in piercings, item 27, and Chromium VI compounds, item 47, of annex XVII) Annex XVII: various ways of limiting, banning or restricting substances, mixtures or groups of substances REACH also sets out the requirement for the information required for safety data sheets. This includes information of hazards, precautions for safe handling and protection (annex II)
Cosmetics Regulation [5]	Based on a weight of evidence approach of all the methods and approaches that is allowed in the cosmetics regulation [18], substances can be subject to restrictions, bans or acceptance in cosmetic products The regulation contains: List of substances prohibited in cosmetic products (annex II). List of substances which cosmetic products must not contain except subject to restrictions laid down (annex III). Lists regarding colourants, preservatives and UV filters allowed in products can be found in annexes IV–VI
Biocidal Products Regulation [3]	“Biocidal products shall not be made available on the market or used unless authorised in accordance with this Regulation” (chap. IV, art. 17). Annex‐I contains a list of approved active substances Biocides can be restricted to professional sale only, have labelling requirements and packaging specifications based on CLP classifications and exposure scenarios [19]
Toys Directive [15]	The Toys directive contains both a list of prohibited allergenic substances and labelling requirements (annexes II, III, 11)
Detergents Regulation [14]	If perfumes are added at concentrations exceeding 0.01% by weight, the allergenic fragrances that appear on the list of substances in annex III of the cosmetic regulation, this must be labelled on the packaging (annex VII, A and art. 13). Preservatives shall also be listed on detergents irrespective of concentration (annex VII, A). Manufacturers shall make available on a website the ingredient data sheet (annex VII, D)
Plant Protection Products [4]	“A plant protection product shall not be placed on the market or used unless it has been authorised in the Member State concerned in accordance with this Regulation” (chap. II, sec. 1, subsec. 1, art. 28). A list of approved active substances found in: “Commission Implementing Regulation (EU) No. 540/2011 of 25 May 2011 implementing Regulation (EC) No. 1107/2009 of the European Parliament and of the Council as regards the list of approved active substances”

The different legislation generally has the same overall goals: ensuring a high level of protection of human health and securing the functioning of the internal European market. Further, REACH also aims to increase competitiveness and innovation (Table 1A).

A wide range of tests for skin sensitization can be used within REACH and the Biocidal Products Regulations (Table 1C). The CLP contains no test requirements but relies on data required under other regulations (CLP Articles 5 and 8). Instead, the CLP provide the criteria for how chemicals should be classified according to several hazard classes, including sensitising properties. The available test results are compared to the criteria in the CLP. If a criterion is fulfilled, the chemical will be classified, and it will have to carry the corresponding warning labelling.

The Local Lymph Node Assay (LLNA) is the first‐choice method for in vivo testing in REACH and the Biocidal Products Regulation and is obligatory under the regulation for Plant Protection Products. In contrast, these tests are prohibited for cosmetic ingredients unless they have been performed before the ban on animal testing. Data from induction experiments in humans (historical) can be used in REACH and Biocidal Products Regulation, as well as the Cosmetics Product Regulation [18]. New Approach Methods (NAMs) can be used under REACH, Cosmetics Regulation [18], and the Biocidal Products Regulation and, if the applicant can justify the need, in the Plant Protection Products Regulation (Table 1C). The tests are in all legislation performed on single substances not the finished mixture or product.

Concerning risk management for skin sensitizers, the CLP and REACH work through providing information on labelling, warnings, restrictions and safety data sheets (SDS). SDS are required, for example, when a substance is classified as hazardous according to the CLP, or under specific conditions such as being categorised as a skin sensitizer under CLP [1] (REACH art. 31). The CLP establishes rules and criteria for classifying skin sensitizers into hazard categories 1, 1B and 1A, which require labelling with the signal word “Warning”, hazard statement H317 and the respective skin sensitising category. The generic concentration limits requiring classification of mixtures as skin sensitising are ≥ 1% for Category 1 and 1B, and ≥ 0.1% for Category 1A. Lower specific concentration limits for classification are set when the generic limits may be insufficiently protective. At concentrations above 10% of the respective classification limit (i.e., 0.1% and 0.01%), mixtures are required to contain the supplemental hazard label EUH208 (also termed ‘elicitation limit’) instead. Category 1 is used if it is not possible to sub‐categorise (Table 1D). Under REACH, it is possible to specifically target individual chemicals and their effects, which is done for nickel and chromium VI in cement and leather [1].

The Cosmetic Regulation works through prohibition (annex II), restrictions (annex III), and positive lists of allowed substances for some types of ingredients such as colourants, preservatives, and UV filters (annexes IV–VI). Since 1997 full ingredient labelling, except for fragrance ingredients, has been required for cosmetics [20]. In 2004, a new rule came into effect, requiring that the identity of a selection of 26 fragrance allergens were to be included in the list of ingredients on the products if the substance is present in concentrations above 0.001% in leave‐on products and above 0.01% in rinse‐off products [21]. The detergents legislation requires that allergenic fragrances, above the concentration of 0.01%, and preservatives, irrespectively of concentration, shall be listed on the ingredients label. For plant protection products and biocidal products, both the active substances and the finished product needs to be approved at the EU level and to be authorised by member states before being placed on the market. The main findings are summarised in Table 2 together with possible actions to improve the protection of the population. This table showcases that the only harmonized topic investigated in this paper is the goal. All the other topics have differences between at least some of the legislations.

**TABLE 2:** Main findings from comparison of legislation and possible actions for improvement.

Topic	Finding examples	Possible action
Goals	All have the goal: High level of protection of human health	Institute feedback mechanisms of effectiveness
Test methods	Wide range: from LLNA is obligatory (PPP) to first choice in vivo (REACH) and not allowed (Cosmetics)	One test strategy/data requirement
Risk assessment models	CLP: Semiquantitative classification^a^ (1A;1B) to unknown (case by case) (Cosmetics)	Develop common quantitative risk assessment methods
Risk management strategies	Wide range from information (CLP) to restriction/bans (Cosmetics Regulation; REACH) and pre‐market approval (PPP; BPR)	Review effectiveness in terms of prevention
Criteria for regulatory action	Differs from case‐by‐case (Cosmetics Regulation) to cut‐off values for classification^b^ (CLP)	Develop harmonised criteria for action

^a^ Categorical division of substances (1, 1B and 1A) based on cut‐off values for specific tests.

^b^ Criteria for 1, 1B and 1A classification based on the size of the problem in relation to exposure.

### 3.2 Questionnaire: Characteristics of Study Population

The recipients were encouraged to share the questionnaires, which were circulated by or within organisations on multiple occasions. Therefore, no precise response frequencies can be calculated. A total of 44 (40%) answers were from regulators and competent authorities, 31 (29%) from researchers, 24 (22%) from industry and 10 (9%) from NGOs.

**FIGURE 1:**
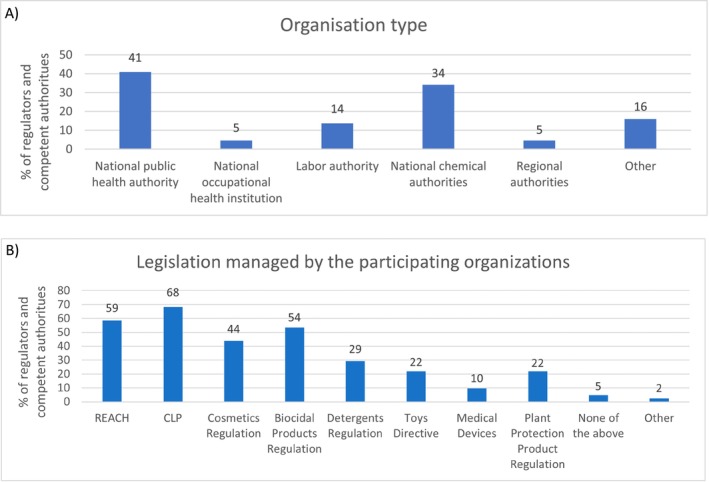
Characterisation of regulators and competent authorities. (A) Shows the percentage of answers given when asked which type of organisation the respondent represents. (B) Shows the legislation managed by the participating regulators and competent authorities as a percentage. Multiple answers were allowed (*n* = 44).

The responses from regulators and competent authorities were distributed across the EU: A total of 78% (21 of 27) were from EU member states and three countries closely related to the EU (Iceland, Norway, and Switzerland), through the European Economic Area or the European single market, adding up to answers from 24 distinct countries. The answers mainly originated from national public health authorities (41%) and national chemical authorities (34%) (Figure 1A). The broad regulations mostly managed by this group were the CLP (59%) and REACH (68%), but all the relevant legislations were represented (Figure 1B). A total of 75% (12/16) of the national public health authorities managed the Cosmetic Products Regulation and 23% (3/13) of the national chemical authorities managed the Cosmetic Products Regulation. A higher percentage of the national public health authorities managed the remaining legislations investigated in this paper than the national chemical authorities (Data S1).

The researchers/clinicians originated from 13 countries, with one representation from Eastern Europe. The respondents indicated to represent universities (23%), hospitals (32%) occupational health institutions (16%), and a mix of other organisations. The participants from the industry consisted of 11 umbrella organisations and 12 individual companies. The individual companies were four small, five medium and three large companies. The industries represented were organisations within the chemical industry, pharmaceutical, employer and business organisations, cosmetics, cleaning and hygiene and paint, coating, and adhesives. Paint, coating and adhesives were the most represented industry branches with 13 respondents. The NGOs were a mix of general consumer protection NGOs (60%) and allergy specific NGOs (40%).

### 3.3 Current Practices: Risk Assessment Methods

Participants who answered that they performed risk assessments were asked to state the risk assessment method they used (Table 3). Only five respondents from NGOs and six from the industry had performed risk assessments of skin sensitizers. Regulators were using mainly risk assessments performing a semiquantitative classification 12/19 (63%). Researchers often used qualitative assessments based on labelling/presence 11/15 (73%), elicitation studies 9/15 (60%) and chemical analysis of products for skin sensitizers 8/15 (53%). Very few have performed a Quantitative Risk Assessment (QRA) (20% of researchers and 10% of regulators) and NGRA had only been done by 21% of regulators (4/19).

**TABLE 3:** Risk assessment methods used by respondents.

Which risk assessment methods do you use?	Regulators (*n* = 19), % (*n*)	Researchers (*n* = 15), % (*n*)	NGOs (*n* = 5), % (*n*)	Industry (*n* = 6), % (*n*)	Total (*n* = 45), % (% range)
Quantitative risk assessment	10% (2)	20% (3)	0	17% (1)	13% (0–20)
Qualitative (hazard) assessment based on labelling/presence	47% (9)	73% (11)	80% (4)	83% (5)	64% (47–83)
Semi‐quantitative classification (e.g., CLP categories 1, 1A, and 1B)	63% (12)	27% (4)	60% (3)	83% (5)	53% (27–83)
Next generation risk assessment	21% (4)	7% (1)	0	0	11% (0–21)
Elicitation studies	37% (7)	60% (9)	0	0	36% (0–60)
Chemical analysis of products for skin sensitizers	N/A	53% (8)	60% (3)	17% (1)	46% (17–60)
Other	26% (5)	7 (1)	0	0	13% (0–26)

*Note*: The number of respondents in each group is shown in the header. N/A indicates that the answer was not available for the group. The respondents could provide multiple answers. Peach colour: Answers above 50% for each group.

Abbreviations: NGO, non‐governmental organisation; NGRA, next generation risk assessment; QRA, quantitative risk assessment.

### 3.4 Views on Level of Protection of the Population

The respondents' views on the level of protection of the general population by current tools and regulations are summarised in Table 4A,B. One third (34%) of regulators, most researchers, and NGOs (77% and 75%, respectively) and a minority of industry (13%) found that the current level of protection was not sufficiently protective for consumers. Very similar results were seen for occupational products except for NGO's where 13% found that regulations were not sufficiently protective. Approximately half (53%) of the national public health authorities answered that the current tools and regulations were adequate regarding consumer products. For the national chemical authorities this number was less than a quarter (23%). Regarding occupational products, the responses by national public health authorities and national chemicals authorities were more alike, with approximately an even share of the respondents who found the current protection adequate (33% and 31%) or insufficient (20% and 31%), respectively (Data S4).

**TABLE 4:** Overall opinion of current tools and regulations of skin sensitizers in EU regarding consumer (A) occupational products (B).

(A) What is your opinion concerning current tools and legislations of skin sensitizers in EU regarding consumer products?	Regulators (*n* = 38), % (*n*)	Researchers (*n* = 30), % (*n*)	NGOs (*n* = 8), % (*n*)	Industry (*n* = 23), % (*n*)	Total (*n* = 99), % (% range)
Current tools and regulations are overprotective	0	7% (2)	0	9% (2)	4% (0–9)
Current tools and regulations are adequate	42% (16)	13% (4)	25% (2)	78% (18)	40% (13–78)
Current tools and regulations are not sufficiently protective	34% (13)	77% (23)	75% (6)	13% (3)	46% (13–75)
Unknown	24% (9)	3% (1)	0	0	10% (0–24)

(B) What is your opinion concerning current tools and regulations of skin sensitizers in EU regarding occupational products?	Regulators (*n* = 38), % (*n*)	Researchers (*n* = 31), % (*n*)	NGOs (*n* = 8), % (*n*)	Industry (*n* = 23), % (*n*)	Total (*n* = 100), % (% range)
Current tools and regulations are overprotective	0	3% (1)	0	9% (2)	3% (0–9)
Current tools and regulations are adequate	34% (13)	10% (3)	49% (4)	78% (18)	38% (10–78)
Current tools and regulations are not sufficiently protective	26% (10)	77% (24)	13% (1)	13% (3)	38% (13–77)
Unknown	40% (15)	10% (3)	38% (3)	0	21% (0–39)

*Note*: The number of respondents in each group is shown in the headers. Peach colour: Answers above 50% for each group.

Abbreviation: NGO, non‐governmental organisation.

**FIGURE 2:**
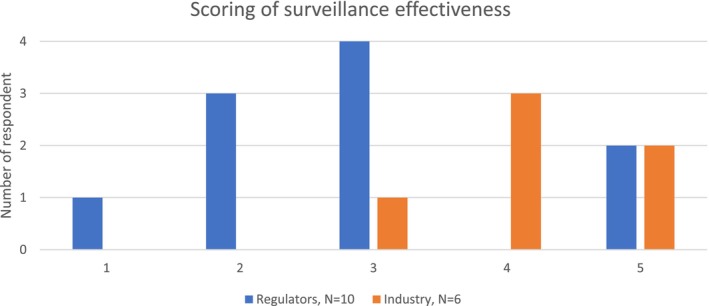
Scoring of surveillance effectiveness regarding the future protection against skin sensitizers. The respondents were asked to score how effective they think their surveillance system is to protect against future skin sensitization on a scale from 1 to 5 (1 being the worst and 5 being the best). The number of respondents in each group is shown in the legends.

Regulators and industry were asked if they had any surveillance system regarding their area of chemicals/products. They were then asked to score how effective this system was at ensuring better future protection towards skin sensitization. The regulators were scoring the effectiveness of the surveillance system at an average 2.6 (out of 5). The industry was scoring their surveillance feedback systems higher with the most answers rating it 4 out of 5 at an average of 4.2 (Figure 2).

### 3.5 Improvement of Legislation

Respondents in all groups indicated areas for improvements in the legislation. Throughout all groups, the two most chosen suggestions were harmonisation across legislation (83% of total) and data sharing (68% of total) (Table 5). Respondents from the industry indicated that they neither support a lowering of the generic concentration limits, nor a ban on using strong skin sensitizers in products with intended skin contact (0% and 10%, respectively). National chemical health authorities wanted a lowering of concentrations of generic concentrations levels (36%) to a higher degree than chemical authorities (18%). Public Health authorities were more inclined (29%) than national chemical authorities (9%) to indicate that a ban on all (strong) skin sensitizers would be beneficial (Data S3).

**TABLE 5:** Improvements of the legislations concerning risk assessment of skin sensitization.

What may improve the legislation concerning risk assessment of skin sensitization on an EU level?	Regulators (*n* = 34), % (*n*)	Researchers (*n* = 31), % (*n*)	NGOs (*n* = 8), % (*n*)	Industry (*n* = 20), % (*n*)	Total (*n* = 93), % (% range)
Harmonisation across regulations	88% (30)	74% (23)	88% (7)	85% (17)	83% (74–88)
Data sharing	65% (22)	74% (23)	75% (6)	60% (12)	68% (60–75)
Lowering of generic concentration limits	29% (10)	68% (21)	63% (5)	0	39% (0–68)
Ban of all (strong) skin sensitizers with intended skin contact	18% (6)	42% (13)	50% (4)	10% (2)	27% (10–50)
No improvements needed	6% (2)	3% (1)	0	5% (1)	4% (0–6)
Other	9% (3)	29% (9)	0	5% (1)	14% (0–29)

*Note*: The number of respondents in each group is shown in the header. The respondents can provide multiple answers. Peach colour: Answers above 50% for each group.

Abbreviation: NGO, non‐governmental organisation.

In total, 4% answered that no improvements were needed. In the comments, it was suggested to introduce the notion of potency to the legislation of skin sensitizers. It was also mentioned that it should be possible to restrict groups of closely related chemicals. Lastly, it was suggested to implement full ingredient labelling for mixtures, articles, and so forth, as this provides crucial information for consumers, healthcare professionals and workers, and so forth.

### 3.6 Views on the Need for Improvements in Risk Assessment Methodology

The respondents in three of the groups; of regulators, researchers, and NGOs, agreed that a wide range of improvements is needed in the risk assessment methodology in the EU. In total 80% (24/30) of researchers suggested, a need for improvements of non‐animal tests and 97% (29/30) responded that more comprehensive exposure data was needed (Table 6). In comparison 45% (9/20) of respondents from industry found that, non‐animal tests needed improvement. Improvements in the understanding of the mechanisms were the second most answered option with a total of 67%. In total 5% of regulators and 10% of industry found that no improvements were needed in the risk assessment of skin sensitizers (0% in the other groups). The most frequent answer across all groups was the need for more comprehensive exposure data. National chemical authorities indicated that we need more comprehensive exposure data and more data concerning aggregated exposures, to a higher degree than national public health authorities. The reverse trend was seen for risk assessment based on elicitation levels (Data S2).

**TABLE 6:** Improvements in risk assessment of skin sensitizers.

What may improve future risk assessment of skin sensitizers on an EU level, regarding the risk assessment methodology?	Regulators (*n* = 37), % (*n*)	Researchers (*n* = 30), % (*n*)	NGOs (*n* = 8), % (*n*)	Industry (*n* = 20), % (*n*)	Total (*n* = 95), % (% range)
Harmonisation across areas of toxicology	54% (20)	67% (20)	75% (6)	65% (13)	62% (54–75)
Improvements of non‐animal test	68% (25)	80% (24)	63% (5)	45% (9)	66% (45–80)
Improvements in the understanding of the underlying mechanisms of skin sensitization	70% (26)	73% (22)	88% (7)	55% (11)	69% (55–88)
More comprehensive exposure data for skin contact to consumer and/or occupational products	70% (26)	97% (29)	63% (5)	70% (14)	78% (63–97)
More data concerning aggregated exposures	62% (23)	73% (22)	63% (5)	45% (9)	62% (45–73)
Data to account for mixture effects	49% (18)	63% (19)	63% (5)	40% (8)	53% (40–63)
Hazard based assessment	N/A	43% (13)	50% (4)	10% (2)	33% (10–50)
Risk assessment based on elicitation levels	27% (10)	57% (17)	50% (4)	25% (5)	37% (25–57)
No improvements needed	5% (2)	0	0	10% (2)	4% (0–10)
Other	11% (4)	17% (5)	0	10% (2)	12% (0–17)

*Note*: The number of respondents in each group is shown in the header. The respondents can provide multiple answers. N/A indicates that the answer was not available for the group. Peach colour: Answers above 50% for each group.

Abbreviation: NGO, non‐governmental organisation.

The comments of the respondents pointed to additional areas of possible improvements. It was suggested that it would be beneficial to perform risk assessments of groups of chemicals, that we need to look more into the potency of chemicals, and it was emphasised that human data should take precedence over other data, especially in cases with a lot of clinical data.

## 4 Discussion

### 4.1 Protection and Surveillance

In this study, we analysed the current major EU legislations relevant to skin sensitization. Furthermore, in a questionnaire, we investigated the opinions of different stakeholders on EU legislations and practices concerning available methods and the resulting level of protection for the population.

We found a general lack of harmonisation in the EU relevant legislations across most of the investigated areas (Table 2). A notable exception was found in the goals of the individual regulations, that is, to ensure a high level of protection of the population. Considerable data exist on a high prevalence, but also incidence of skin sensitization in the population and subgroups at risk in EU [6]. Therefore, the effectiveness of EU regulations in this area should be questioned. Feedback mechanisms to initiate or evaluate relevant regulations based on current European data on the occurrence of skin sensitization, are limited.

Considering the wide spectrum of interests among respondents, it is noteworthy that overall, 46% and 38% found current regulations not to be sufficiently protective concerning consumer and occupational products, respectively. However, a considerable difference between the groups, for example, researchers (77%) and industry representatives (13%) were evident. This diversity shows that a harmonised surveillance system, able to feedback data on the occurrence of skin sensitization to the regulatory system, should be further considered. Such a system could shed more light on the actual workings of the current regulatory system and contribute to a more consistent assessment of the protectiveness of the legislation. For cosmetic products, companies have a legal obligation to monitor adverse health effects potentially caused by their products on the market through a process known as cosmetovigilance (Cosmetics Regulation art. 23 [5]), while regulators monitor serious undesirable effects and report these through a common rapid alert system [22]. The cosmetovigilance is a passive system, relying on complaints, and does not focus on skin sensitization data [23]. Some countries may also have, or have had, national surveillance systems [24]. In the present study, regulators were asked to score the effectiveness of their surveillance system to ensure better protection on a scale from 1 to 5 (best), on average they scored 2.6, while Industry representatives scored an average of 4, being much more satisfied with the present system. A European Surveillance System on Contact Allergies (ESSCA), collecting and analysing data from patients with skin sensitization and ACD, has been functioning among dermatologists in the EU since 2002 [25]. The network publishes data on specific occupational [26] and non‐occupational [27, 28] skin sensitizers [29], exposures, and temporal trends [30, 31]. It has recently been suggested by the EU Commission to establish a systematic collection of human biomonitoring data generated in the EU to inform policy makers [32]. The ESSCA network or similar could be considered to function as an independent surveillance system and transparent source of feedback concerning the effectiveness of EU regulations in preventing skin sensitization.

### 4.2 Harmonisation of Legislation

All questionnaire groups identified areas in the legislation where improvements were needed. All groups agreed that harmonisation and data sharing were potential areas of improvement (Table 5). This was further substantiated in a recent publication concerning preservatives in non‐cosmetic products. Several examples were given of sensitising preservatives regulated in one area, but not classified as skin sensitizers under CLP [33], for example, the preservative methyldibromo glutaronitrile (MDBGN), which had been permitted in cosmetic products in concentrations up to 0.1% in 1980's, and was banned from all use in cosmetic products in 2007, due to a widespread epidemic of skin sensitization and ACD [34]. As such, MDBGN was not deemed safe at any concentration, but still it does not have a harmonised classification under CLP. Only 57% of 1500 notifications of MDBGN in CLP included Skin Sens. as self‐classification, and none of the notifications with a concentration limit below 1% [33].

The needs for improvement are in line with the strategy ‘one substance, one assessment’ of the Commission adopting three legislative proposals to streamline assessments of chemicals across EU legislations; strengthen the knowledge base on chemicals; and ensure early detection and action on emerging chemical risks (Dec. 2023) [32].

### 4.3 Risk Assessment Challenges

One of the major challenges in the area of skin sensitization is that no risk assessment method has been generally accepted, even though the first suggestion of a QRA model for skin sensitization was developed decades ago [35, 36]. The model is based on general principles of risk assessment in toxicology, except that the critical exposure parameter is the dose of skin sensitizer per unit area of skin [37] and not the total dose as in (most) other areas of toxicology. The model was further developed and adopted by the fragrance industry [38]. It has also been investigated in other regulatory areas, such as pesticides [39]. The skin sensitization QRA model has several times been assessed by SCCS and its predecessors, without being formally accepted. The committee found important areas where improvements were needed [40, 41, 42]. This is also reflected in our questionnaires, in that few respondents, for example, regulators (10%) and industry (17%), reported using this risk assessment methodology (Table 3). Moreover, all the stakeholders highlighted that comprehensive exposure data and data to account for aggregate exposures and mixture effects are further areas for improvement. The QRA for skin sensitizers has, as many other toxicological models, relied on data from animal assays to derive a dose level not expected to sensitise. While these are still obligatory under some regulations, for example, the Plant Protection Products Regulation, they are not allowed to be performed in other areas such as cosmetics.

A total of 80% of researchers suggested that improvements in alternatives for animal tests were needed (Table 6). This area is relevant since the EU is aiming to replace animal models with New Approach Methodologies (NAMs) [43]. Although the OECD has described mechanism‐based adverse outcome pathways for skin sensitization and provided guidance for Integrated Approaches to Testing and Assessment (IATAs) [44], as well as describing the related Key Events (1–4) and the corresponding NAMs in the Defined Approaches (DA) [45], challenges remain concerning the quantitative aspects, effect of real‐life mixtures and translation to safe levels of exposure. A key problem is that most legislations describe methods to assess skin sensitization, but many of them do not clearly define the decisions and consequences that should apply once a substance or product is identified as a sensitizer. Through CLP, the classification of a substance as a skin sensitizer is made on specific cut‐off values applied to data derived from tests such as LLNA and epidemiological data (e.g., tabs. 3.4.3 and 3.4.4 in CLP [2]). No such clear criteria and instructions are present to describe when substances are adopted into the different annexes of, for example, the Cosmetic Products Regulation. The accepted tests can be performed on a case‐by‐case basis and used in a weight of evidence approach [18]. This could be seen as a major deficit in relation to the goals set to prevent skin sensitization and protect human health. This problem is not exclusive to the EU. In 2017, substantial differences and lacking information were identified worldwide regarding the accepted methods used for regulatory purposes. It was also noted that the endpoints of these tests ranged from hazard classification to risk assessment [46]. While hazard classification provides valuable information about a chemical's intrinsic hazards, it is important to remember that the risk towards skin sensitization depends on the potency of the chemical and the actual exposure [47]. Risk assessment should cover a broader range skin sensitizers across various use scenarios. For this to be effective, many issues identified in this paper require further attention. Specifically, as highlighted in our questionnaire, a lack of data on different exposure scenarios, aggregated exposures and mixture effects, hampers effective risk assessment.

### 4.4 Limitations

Our study has some important limitations. The overall number of respondents to the questionnaires is small, particularly from NGOs and Industry. Therefore, it is uncertain how representative the results from these groups are. However, the results still show some trends. Through free text comments, additional information was gathered. This was however mostly used by researchers and regulators. Furthermore, although the questionnaires were developed fully *denovo*, tested internally multiple times and three times sent out to target groups for testing, it cannot be excluded that some questions could have been misinterpreted, however, no indication of this was seen.

## 5 Conclusions and Recommendations

The lack of specific guidance concerning protection against skin sensitization in most of the reviewed major regulations in this study hampers effective prevention of skin sensitization in the European population. In line with this, a significant part of stakeholders in the questionnaires found that current regulations were not sufficiently protective against skin sensitization and should be improved. Based on the results from the questionnaires, it is recommended to harmonise and develop all relevant regulations to provide specific guidance concerning (quantitative) risk assessment of skin sensitizers. For this, more and better data are needed, in particular with regard to exposure monitoring and modelling. Furthermore, there is a need for developing NAMs for more reliable identification of skin sensitizers and better information concerning the presence of skin sensitizers in all types of products via for example full ingredient labelling. Lastly, systematic, independent and transparent monitoring of skin sensitization in the EU should be considered as a source of feedback on the effectiveness of EU regulations to prevent skin sensitization.

## Author Contributions

**Mathias Krogh Pedersen:** conceptualization, writing – original draft, writing – review and editing, project administration, formal analysis, data curation, investigation, software. **Jakob Ferløv Baselius Schwensen:** writing – review and editing, conceptualization, supervision. **Jose Hernán Alfonso:** writing – review and editing, conceptualization. **Steen Mollerup:** conceptualization, writing – review and editing. **Gianluca Selvestrel:** conceptualization, writing – review and editing. **Christina Rudén:** conceptualization, writing – review and editing. **Martin F. Wilks:** conceptualization, writing – review and editing. **Jeanne Duus Johansen:** conceptualization, writing – original draft, writing – review and editing, supervision, funding acquisition.

## Conflicts of Interest

Jose H. Alfonso has received an unrestricted research grant from Sanofi. The other authors have no conflicts of interest to declare.

## Supporting information


**Data S1.** Legislation managed by National public health authorities and national chemical authorities. The number of respondents from each group is shown in the legends. Multiple answers were allowed. Only participants who only managed either National public health authorities or national chemical authorities is shown.
**Data S2**. Improvements of risk assessment. The number of respondents from each group is shown in the legends. Multiple answers were allowed. Only participants who only managed either National public health authorities or national chemical authorities are shown.
**Data S3**. Improvements of legislation. The number of respondents from each group is shown in the legends. Multiple answers were allowed. Only participants who only managed either National public health authorities or national chemical authorities is shown.
**Data S4**. Opinion of overall state of current protection of occupational (A) and consumer products (B). The number of respondents from each group is shown in the legends. Only participants who only managed either National public health authorities or national chemical.

**Data S5.** Questionnaire for Industry.

**Data S6.** Questionnaire for competent authorities and regulators Questionnaire concerning skin sensitising chemicals.

**Data S7.** Questionnaire for researchers and clinicians Questionnaire concerning skin sensitising chemicals.

**Data S8.** Questionnaire for NGOs.

## Data Availability

Research data are not shared.
