# Supplementary material for: Legislation and Current Practices Concerning Risk Assessment of Skin Sensitizers in the European Union: A Comparative and Survey Study
Source: Contact Dermatitis. 2025 Feb 7;92(6):446–59. doi: 10.1111/cod.14754 (PMC12055314; doi:10.1111/cod.14754)
Supplement: Supplementary file 1 — Data S1. Legislation managed by National public health authorities and national chemical authorities. The number of respondents from each group is shown in the legends. Multiple answers were allowed. Only participants who only managed either National public health authorities or national chemical authorities is shown. Data S2. Improvements of risk assessment. The number of respondents from each group is shown in the legends. Multiple answers were allowed. Only participants who only managed either National public health authorities or national chemical authorities are shown. Data S3. Improvements of legislation. The number of respondents from each group is shown in the legends. Multiple answers were allowed. Only participants who only managed either National public health authorities or national chemical authorities is shown. Data S4. Opinion of overall state of current protection of occupational (A) and consumer products (B). The number of respondents from each group is shown in the legends. Only participants who only managed either National public health authorities or national chemical. [file COD-92-446-s005.docx]

# Supplementary

***S1 Legislations managed by National public health authorities and national chemical authorities.*** *The number of respondents from each group is shown in the legends. Multiple answers were allowed. Only participants who only managed either National public health authorities or national chemical authorities is shown.*

***S2. Improvements of risk assessment.*** *The number of respondents from each group is shown in the legends. Multiple answers were allowed. Only participants who only managed either National public health authorities or national chemical authorities are shown.*

***S3. Improvements of legislations.*** *The number of respondents from each group is shown in the legends. Multiple answers were allowed. Only participants who only managed either National public health authorities or national chemical authorities is shown.*

A)

*B)*

***S4. Opinion of overall state of current protection of occupational (A) and consumer products (B).*** *The number of respondents from each group is shown in the legends. Only participants who only managed either National public health authorities or national chemical*
