# Supplementary material for: Legislation and Current Practices Concerning Risk Assessment of Skin Sensitizers in the European Union: A Comparative and Survey Study
Source: Contact Dermatitis. 2025 Feb 7;92(6):446–59. doi: 10.1111/cod.14754 (PMC12055314; doi:10.1111/cod.14754)
Supplement: Supplementary file 2 — Data S5. Questionnaire for Industry. [file COD-92-446-s002.pdf]

S5. Questionnaire for Industry

Questionnaire concerning skin sensitizing chemicals

Thank you for agreeing to participate in this study.

Your answers will be used in scientific publications and to improve future risk assessments of skin sensitizers across the European Union. This project is co-funded by the European Union through the European Partnership for the Assessment of Risk from Chemicals (PARC).

If you have any questions or concerns, please feel free to contact us through the contact information provided in the E-mail/formula through which we contacted you. By entering data or clicking "Next Page" in the questionnaire you agree that we can use the provided information for the mentioned purposes.

Please state which country your organization belongs to, the name of your organization, your job title and your name:

Country: ________________________

Organization: ________________________

Job title of respondent: ________________________

Name: ________________________

Is your organization an employers' organization or association representing the interest of multiple companies/industries?

◯ Yes
◯ No

How many people do you have employed in your manufacturing section?

◯ Fewer than 10
◯ 10-49
◯ 50-249
◯ 250 or above
◯ Unknown

Please indicate your company's annual turnover rate in EUR:

◯ Below 2 million €
◯ Between 2 to 10 million €
◯ Between 10 to 50 million €
◯ Over 50 million €
◯ Unknown

Please indicate your company's annual balance sheet total in EUR:

◯ Below 2 million €
◯ Between 2 to 10 million €
◯ Between 10 to 43million €
◯ Over 43 million €
◯ Unknown

Have you received training in risk assessment?

◯ Yes
◯ no

Is allergy and sensitizing chemicals a focus area of your organization?

◯ Yes
◯ No

What is your opinion concerning current tools and regulations of skin sensitizers in EU regarding consumer products?	◯ Current tools and regulations are overprotective<br>◯ Current tools and regulations are adequate<br>◯ Current tools and regulations are not sufficiently protective<br>◯ Unknown
What is your opinion concerning current tools and regulations of skin sensitizers in EU regarding occupational products?	◯ Current tools and regulations are overprotective<br>◯ Current tools and regulations are adequate<br>◯ Current tools and regulations are not sufficiently protective<br>◯ Unknown
Does your organization take part in/perform risk assessments of chemicals/mixtures/products regarding its skin sensitizing properties?	◯ Yes<br>◯ No<br>◯ Unknown
Which method(s) do you use?<br>(Select all that apply)	☐ Assessment based on ingredient labelling<br>☐ Chemical analysis of products for skin sensitizers<br>☐ Classification (e.g., GHS/CLP categories 1, 1A, and 1B)<br>☐ Elicitation/patient studies<br>☐ Next Generation Risk Assessments (NGRA) using only New Approach Methods / Non-animal methods<br>☐ Dermal sensitization quantitative risk assessment (QRA) as for fragrance ingredients (as described by SCCS 2008) or the revised version QRA2 (as described by SCCS 2017)<br>☐ Other<br>☐ We do not perform risk assessments
Other, please specify:	__________________________________
How do you account for mixture effects in the risk assessment of skin sensitizers in mixtures? (select all that apply)	☐ We use a mixture assessment factor (or mixture allocation factor) - if possible, please enter the value:<br>☐ We assume additive effects in mixtures of skin sensitizers<br>☐ We do not take the mixture effect into account<br>☐ Other<br>☐ Comments:
Please, specify correction factor:	__________________________________
Comments:	____________________________
Other, please specify:	__________________________________

Do you yourself generate data for the purpose of risk assessments? (Select all that apply)	☐ In vivo(human)<br>☐ In vivo(animal)<br>☐ In vitro<br>☐ In chemico<br>☐ In silico<br>☐ A combination of in chemico and in vitro e.g., for use in Defined Approaches as described by OECD guideline No. 497 (2021 OECD Guidelines for the Testing of Chemicals)<br>☐ no

Further questions about risk assessment:

Do you perform "In-house" sensitization test or do you obtain the data from external bodies? (Select all that apply)

- ☐ In-house
- ☐ By external bodies
- ☐ Unknown

Have you ever performed in-house Local Lymph Node Assays?

- ◯ Yes, and we are willing to share protocols and/or data (possibility to attach files/links).
- ◯ Yes, but we cannot share these methods or results
- ◯ No

Feel free to upload relevant files here:

Feel free to provide relevant links or information here:

____

Does your organization have a monitoring system concerning consumer and/or occupational complaints of skin sensitization related to your organization and its related products?

- ◯ Yes
- ◯ No
- ◯ Unknown

Which kind of data do you collect?

- ☐ Consumer complaints from cosmetics
- ☐ Consumer complaints from detergents
- ☐ Consumer complaints (other products)
- ☐ Workers' complaints
- ☐ Other, please specify:

Other, please specify:

____

How are you collecting the surveillance data?

- ◯ The monitoring system is a passive system, where you receive complaints
- ◯ The monitoring system is an active system, where you engage in surveys or a sentinel surveillance system etc.
- ◯ Other, please specify:

Other, please specify:

____

Is there a feedback mechanism in place that actively aims to ensure better protection against skin sensitization?

- ◯ Yes
- ◯ No
- ◯ Unknown

Please score how effective you think the monitoring system/feed-back mechanism is at preventing future skin sensitization? (5 being very effective and 1 being ineffective)

- ◯ 1
- ◯ 2
- ◯ 3
- ◯ 4
- ◯ 5
- ◯ Unknown

What may improve the regulation concerning risk assessment of skin sensitization on an EU level?<br>(Select all that apply)	☐ Harmonization across regulations<br>☐ Data sharing<br>☐ Lowering of generic concentration limits of components of a mixture that leads to classification as skin sensitizer (Category 1, 1A, and 1B) in CLP<br>☐ Raising of generic concentration limits of components of a mixture that leads to classification as skin sensitizer (Category 1, 1A, and 1B) in CLP<br>☐ Ban of all (strong) skin sensitizers with intended skin contact<br>☐ No improvements needed<br>☐ Other<br>☐ Comments:
Other, please specify:	__________________________________
Comments:	__________________________________
What may improve future risk assessment of skin sensitizers on an EU level, regarding the risk assessment methodology?<br>(Select all that apply)	☐ Harmonization across areas of toxicology<br>☐ Improvements of non-animal test<br>☐ Improvements in the understanding of the underlying mechanisms of skin sensitization<br>☐ More comprehensive exposure data for skin contact to consumer and/or occupational products<br>☐ More data concerning aggregated exposures<br>☐ Data to account for mixture effects<br>☐ Hazard based assessment<br>☐ Risk assessment based on elicitation levels<br>☐ No improvements needed<br>☐ Other, please specify:<br>☐ Comments:
Other, please specify:	__________________________________
Comments:	___________________________
May we contact you for further information?	◯ Yes<br>◯ No
Yes selected, please specify:	
Email:	___________________________
Thank you for participating.<br>Feel free to leave any comments:	__________________________________
