# Supplementary material for: Legislation and Current Practices Concerning Risk Assessment of Skin Sensitizers in the European Union: A Comparative and Survey Study
Source: Contact Dermatitis. 2025 Feb 7;92(6):446–59. doi: 10.1111/cod.14754 (PMC12055314; doi:10.1111/cod.14754)
Supplement: Supplementary file 3 — Data S6. Questionnaire for competent authorities and regulators Questionnaire concerning skin sensitising chemicals. [file COD-92-446-s001.pdf]

S6. Questionnaire for competent authorities and regulators

Questionnaire concerning skin sensitizing chemicals

Thank you for agreeing to participate in this study!

Your answers will be used in scientific publications and to improve future risk assessments of skin sensitizers across the European Union. This project is co-funded by the European Union through the European Partnership for the Assessment of Risk from Chemicals (PARC).

If you have any questions or concerns, please feel free to contact us through the contact information provided in the E-mail/formula through which we contacted you. By entering data or clicking "Next Page" in the questionnaire you agree that we can use the provided information for the mentioned purposes.

Please state which country your organization belongs to, the name of the organization and the job title of the respondent

---

Country: ______________________

Organization: ______________________

Job title of respondent: ______________________

Which kind organization do you represent? (Select all that apply)

- ☐ National public health authority
- ☐ National occupational health institution
- ☐ Labor authority
- ☐ National chemical authorities
- ☐ Regional authorities
- ☐ Environmental protection authorities
- ☐ EU bodies
- ☐ ECHA
- ☐ EEA
- ☐ Other

Other, please specify ______________________

Does your organization take an interest in regulations/legislations of skin sensitizing chemicals/mixtures or risk assessment of skin sensitizers?

- ◯ Yes
- ◯ No

Whom can we contact for information about this topic?

Organization: ______________________

Contact Person: ______________________

E-mail: ______________________

Which of the following, if any, EU regulations/legislations do you manage, regarding skin sensitizers?<br>(Select all that apply)	☐ REACH<br>☐ CLP<br>☐ Cosmetics Regulation<br>☐ Biocidal Products Regulation<br>☐ Detergents Regulation<br>☐ Toys Directive<br>☐ Medical Devices<br>☐ Plant Protection Product Regulation<br>☐ None of the above<br>☐ Other
Other, please specify:	__________________________
Does your organization take part in risk assessments of chemicals/mixtures regarding its skin sensitizing properties?	○ Yes<br>○ No<br>○ Unknown
Which method(s) do you use?<br>(Select all that apply)	☐ Dermal sensitization quantitative risk assessment (QRA) for fragrance ingredients (as described by SCCS 2008) or the revised version QRA2 (as described by SCCS 2017)<br>☐ Qualitative assessment based on labelling/presence only<br>☐ Semi-quantitative classification (e.g., GHS/CLP categories 1, 1A, and 1B)<br>☐ Next Generation Risk Assessments (NGRA) using only New Approach Methods / Non-animal methods<br>☐ Elicitation studies<br>☐ Other<br>☐ We do not perform risk assessments
Other, please specify:	__________________________
How do you correct for mixture effects in the risk assessment of skin sensitizers in mixtures?	○ We use a correction factor - if possible, please enter the value:<br>○ We assume additive effects in mixtures of skin sensitizers<br>○ We do not take the mixture effect into account<br>○ Other
Please, specify correction factor:	__________________________
Other, please specify:	__________________________
Does your organization have a monitoring system concerning consumer and/or occupational complaints of skin sensitization related to your organization and its related products?	○ Yes<br>○ No<br>○ Unknown

Which kind of data do you collect?<br>(Select all that apply)	☐ Consumer complaints from cosmetics<br>☐ Consumer complaints from detergents<br>☐ Consumer complaints (other products)<br>☐ Workers' complaints<br>☐ Other
Other, please specify:	__________________________________
How are you collecting the surveillance data?	◯ The monitoring system is a passive system, where you receive complaints<br>◯ The monitoring system is an active system, where you engage in surveys or a sentinel surveillance system etc.<br>◯ Other
Other, please specify:	__________________________________
Is there a feedback mechanism in place that actively aims to ensure better protection against skin sensitization?	◯ Yes<br>◯ No<br>◯ Unknown
Please score how effective you think the monitoring system/feed-back mechanism is at preventing future skin sensitization?<br>(5 being very effective and 1 being ineffective)	◯ 5<br>◯ 4<br>◯ 3<br>◯ 2<br>◯ 1<br>◯ Unknown
What is your opinion concerning current tools and regulations of skin sensitizers in EU regarding occupational products?	◯ Current tools and regulations are overprotective<br>◯ Current tools and regulations are adequate<br>◯ Current tools and regulations are not sufficiently protective<br>◯ Unknown
What is your opinion concerning current tools and regulations of skin sensitizers in EU regarding consumer products?	◯ Current tools and regulations are overprotective<br>◯ Current tools and regulations are adequate<br>◯ Current tools and regulations are not sufficiently protective<br>◯ Unknown
What may improve the regulation concerning risk assessment of skin sensitization on an EU level?<br>(Select all that apply)	☐ Harmonization across regulations<br>☐ Data sharing<br>☐ Lowering of generic concentration limits of components of a mixture that leads to classification as skin sensitizer (Category 1, 1A, and 1B) in CLP<br>☐ Risk assessment based on elicitation levels<br>☐ No improvements needed<br>☐ Other<br>☐ Comments:
Other, please specify:	__________________________________

Comments:

____

What may improve future risk assessment of skin sensitizers on an EU level, regarding the risk assessment methodology?
(Select all that apply)

- ☐ Harmonization across areas of toxicology
- ☐ Improvements of non-animal test
- ☐ Improvements in the understanding of the underlying mechanisms of skin sensitization
- ☐ More comprehensive exposure data for skin contact to consumer and/or occupational products
- ☐ More data concerning aggregated exposures
- ☐ Data to correct for mixture effects
- ☐ Ban of all (strong) skin sensitizers
- ☐ No improvements needed
- ☐ Other, please specify:
- ☐ Comments:

Other, please specify:

____

Comments:

____

May we contact you for further information?

- ◯ Yes
- ◯ No

Yes selected, please specify:

Organization:

____

Contact person:

____

Email:

____

Thank you for participating.
Feel free to leave any comments:

____

This questionnaire is intended for regulatory and competent authorities across EU member nations. If your organization works with risk assessment, surveillance, or regulatory functions of skin sensitizers, please go back, and press yes.
Otherwise, thanks for your attention, but you are not eligible to continue this questionnaire.
