# Supplementary material for: Legislation and Current Practices Concerning Risk Assessment of Skin Sensitizers in the European Union: A Comparative and Survey Study
Source: Contact Dermatitis. 2025 Feb 7;92(6):446–59. doi: 10.1111/cod.14754 (PMC12055314; doi:10.1111/cod.14754)
Supplement: Supplementary file 4 — Data S7. Questionnaire for researchers and clinicians Questionnaire concerning skin sensitising chemicals. [file COD-92-446-s003.pdf]

S7. Questionnaire for researchers and clinicians
Questionnaire concerning skin sensitizing chemicals

Thank you for agreeing to participate in this study!

Your answers will be used in scientific publications and to improve future risk assessments of skin sensitizers across the European Union. This project is co-funded by the European Union through the European Partnership for the Assessment of Risk from Chemicals (PARC).

If you have any questions or concerns, please feel free to contact us through the contact information provided in the E-mail/formula through which we contacted you. By entering data or clicking "Next Page" in the questionnaire you agree that we can use the provided information for the mentioned purposes.

Please state which country your organization belongs to, the name of your organization, your job title and your name:

Country: ______________________

Organization: ______________________

Job title of respondent: ______________________

Name: ______________________

Do you perform clinical or basic research regarding allergic contact dermatitis or skin sensitization?

- ◯ Yes
- ◯ No

This questionnaire is intended for researchers across EU member nations. If your research involves allergic contact dermatitis, please go back, and press yes.
Otherwise, thanks for your attention, but you are not eligible to continue this questionnaire.

Do you collect data concerning patient cases or epidemiological data of skin sensitization related to products?

- ◯ Yes
- ◯ No
- ◯ Unknown

Which kind of data do you collect? (Select all that apply)

- ☐ Patient cases/epidemiological data with cosmetic related dermatitis
- ☐ Patient cases/epidemiological data with detergent related dermatitis
- ☐ Patient cases/epidemiological data from occupational skin diseases
- ☐ Other patient cases/epidemiological data regarding skin sensitization
- ☐ Other

Other, please specify: ______________________

On which level are you collecting patient data? (Select all that apply)

- ☐ From our own clinic/hospital
- ☐ Regional data
- ☐ National data
- ☐ On an EU level
- ☐ Other

Other, please specify:	__________________________________
Do you share the aggregated results with national and/or EU authorities?	◯ Yes<br>◯ No<br>◯ Unknown
Please score how effective you think the data sharing is at preventing future skin sensitization? (5 being very effective and 1 being ineffective)	◯ 5<br>◯ 4<br>◯ 3<br>◯ 2<br>◯ 1<br>◯ Unknown
Do you cooperate with national authorities and/or competent authorities to improve the regulation of skin sensitizers?	◯ Yes, to a high degree (e.g. participation in committee meetings, frequent meetings or contact)<br>◯ Yes, to some degree (occasional contact)<br>◯ no<br>◯ Unknown
Do you cooperate with national authorities, competent authorities, industry, and/or NGOs to increase awareness of skin sensitizers?	◯ Yes, to a high degree<br>◯ Yes, to some degree<br>◯ no<br>◯ Unknown
Do you find that researchers can influence regulations of sensitizing chemicals in a sufficient way?	◯ Yes, to a high degree<br>◯ Yes, to some degree<br>◯ no<br>◯ Unknown
What is your opinion concerning current tools and regulations of skin sensitizers in EU regarding consumer products?	◯ Current tools and regulations are overprotective<br>◯ Current tools and regulations are adequate<br>◯ Current tools and regulations are not sufficiently protective<br>◯ Unknown
What is your opinion concerning current tools and regulations of skin sensitizers in EU regarding occupational products?	◯ Current tools and regulations are overprotective<br>◯ Current tools and regulations are adequate<br>◯ Current tools and regulations are not sufficiently protective<br>◯ Unknown
What may improve the regulation concerning risk assessment of skin sensitization on an EU level? (Select all that apply)	☐ Harmonization across regulations<br>☐ Data sharing<br>☐ Lowering of generic concentration limits of components of a mixture that leads to classification as skin sensitizer (Category 1, 1A, and 1B) in CLP<br>☐ Ban of all (strong) skin sensitizers with intended skin contact<br>☐ No improvements needed<br>☐ Other<br>☐ Comments:
Other, please specify:	____

Comments:	__________________________________
What may improve future risk assessment of skin sensitizers on an EU level, regarding the risk assessment methodology?<br>(Select all that apply)	☐ Harmonization across areas of toxicology<br>☐ Improvements of non-animal test<br>☐ Improvements in the understanding of the underlying mechanisms of skin sensitization<br>☐ More comprehensive exposure data for skin contact to consumer and/or occupational products<br>☐ More data concerning aggregated exposures<br>☐ Data to account for mixture effects<br>☐ Hazard based assessment<br>☐ Risk assessment based on elicitation levels<br>☐ No improvements needed<br>☐ Other, please specify:<br>☐ Comments:
Other, please specify:	__________________________________
Comments:	__________________________________
Do you think that the EU is efficient at protecting against skin sensitization?	○ Yes, to a high degree<br>○ Yes, to some degree<br>○ no<br>○ Unknown
Do you think that skin sensitization as a subject gets enough attention in EU?	○ Yes, to a high degree<br>○ Yes, to some degree<br>○ no<br>○ Unknown
Do you take part in/perform risk assessments of chemicals/mixtures regarding its skin sensitizing properties?	○ Yes<br>○ No<br>○ Unknown
Which method(s) do you use?<br>(Select all that apply)	☐ Assessment based on ingredient labelling<br>☐ Chemical analysis of products for skin sensitizers<br>☐ Classification (e.g., GHS/CLP categories 1, 1A, and 1B)<br>☐ Elicitation/patient studies<br>☐ Next Generation Risk Assessments (NGRA) using only New Approach Methods / Non-animal methods<br>☐ Dermal sensitization quantitative risk assessment (QRA) as for fragrance ingredients (as described by SCCS 2008) or the revised version QRA2 (as described by SCCS 2017)<br>☐ Other<br>☐ We do not perform risk assessments
Other, please specify:	__________________________________

How do you correct for mixture effects in the risk assessment of skin sensitizers in mixtures?	☐ We use a mixture assessment factor (or mixture allocation factor) - if possible, please enter the value:<br>☐ We assume additive effects in mixtures of skin sensitizers<br>☐ We do not take the mixture effect into account<br>☐ Other<br>☐ Comments:
Please, specify correction factor:	__________________________________
Comments:	__________________________________
Other, please specify:	__________________________________
Do you yourself generate data for the purpose of risk assessments? (Select all that apply)	☐ In vivo(human)<br>☐ In vivo(animal)<br>☐ In vitro<br>☐ In chemico<br>☐ In silico<br>☐ A combination of in chemico and in vitro e.g., for use in Defined Approaches as described by OECD guideline No. 497 (2021 OECD Guidelines for the Testing of Chemicals)<br>☐ no
Do you perform "In-house" sensitization test or do you obtain the data from external bodies? (Select all that apply)	☐ In-house<br>☐ By external bodies<br>☐ Unknown
Have you ever performed in-house Local Lymph Node Assays?	☐ Yes, and we are willing to share protocols and/or data (possibility to attach files/links).<br>☐ Yes, but we cannot share these methods or results<br>☐ No
Feel free to upload relevant files here:	
Feel free to provide relevant links or information here:	__________________________________
May we contact you for further information?	◯ Yes<br>◯ No
Yes selected, please specify:	__________________________________

Email:

Thank you for participating.
Feel free to leave any comments:
