# Supplementary material for: Legislation and Current Practices Concerning Risk Assessment of Skin Sensitizers in the European Union: A Comparative and Survey Study
Source: Contact Dermatitis. 2025 Feb 7;92(6):446–59. doi: 10.1111/cod.14754 (PMC12055314; doi:10.1111/cod.14754)
Supplement: Supplementary file 5 — Data S8. Questionnaire for NGOs. [file COD-92-446-s004.pdf]

S8. Questionnaire for NGOs

Questionnaire concerning skin sensitizing chemicals

Thank you for agreeing to participate in this study!

Your answers will be used in scientific publications and to improve future risk assessments of skin sensitizers across the European Union. This project is co-funded by the European Union through the European Partnership for the Assessment of Risk from Chemicals (PARC).

If you have any questions or concerns, please feel free to contact us through the contact information provided in the E-mail/formula through which we contacted you. By entering data or clicking "Next Page" in the questionnaire you agree that we can use the provided information for the mentioned purposes.

Please state which country your organization belongs to, the name of the organization and the job title of the respondent

---

Country: ______________________________

Organization: ______________________________

Job title of respondent: ______________________________

---

2. Does your organization take an interest in any of the following subjects regarding skin sensitizers:
- Regulations/legislations of skin sensitizing chemicals/mixtures
- Risk assessment
- Hazard assessment
- Analysis of products
- Providing information to consumers/workers

○ Yes
○ No

This questionnaire is intended for NGOs across EU member nations. If your organization works with or take an interest in risk assessment, surveillance, information sharing on skin sensitizers, or regulation of skin sensitizers etc. please go back and press yes. You are also welcome to look at the next questions for relevance to your NGO. Otherwise, thanks for your attention, but you are not eligible to continue this questionnaire.

In which way, are you showing interest?

☐ Politically by focusing on the problem, addressing politicians etc.
☐ Performing tests/investigations of skin sensitizers in consumer/occupational products or chemicals
☐ Publishing information, leaflets, films etc.
☐ Providing tools for consumers e.g., apps with information on how to avoid skin sensitizers
☐ Providing tools for workers e.g., apps with information on how to avoid skin sensitizers
☐ Unknown
☐ Other

Other, please specify: ______________________________

Do you have a monitoring system concerning consumer and/or occupational complaints of skin sensitization related to products?

○ Yes
○ No
○ Unknown

Which kind of data do you collect?<br>(Select all that apply)	☐ Consumer complaints from cosmetics<br>☐ Consumer complaints from detergents<br>☐ Consumer complaints (other products)<br>☐ Workers' complaints<br>☐ Other
Other, please specify:	__________________________________
How are you collecting the surveillance data?	◯ The monitoring system is a passive system, where you receive complaints<br>◯ The monitoring system is an active system, where you engage in surveys or a sentinel surveillance system etc.<br>◯ Other
Other, please specify:	__________________________________
Is there a feedback mechanism in place that actively aims to ensure better protection against skin sensitization?	◯ Yes<br>◯ No<br>◯ Unknown
Please score how effective you think the monitoring system/feed-back mechanism is at preventing future skin sensitization?<br>(5 being very effective and 1 being ineffective)	◯ 5<br>◯ 4<br>◯ 3<br>◯ 2<br>◯ 1<br>◯ Unknown
What is your opinion concerning current tools and regulations of skin sensitizers in EU regarding occupational products?	◯ Current tools and regulations are overprotective<br>◯ Current tools and regulations are adequate<br>◯ Current tools and regulations are not sufficiently protective<br>◯ Unknown
What is your opinion concerning current tools and regulations of skin sensitizers in EU regarding consumer products?	◯ Current tools and regulations are overprotective<br>◯ Current tools and regulations are adequate<br>◯ Current tools and regulations are not sufficiently protective<br>◯ Unknown
What may improve the regulation of skin sensitizers on an EU level? (Select all that apply)	☐ Harmonization across regulations<br>☐ Data sharing<br>☐ Lowering of generic concentration limits of components of a mixture that leads to classification as skin sensitizer (Category 1, 1A, and 1B) in CLP<br>☐ Ban of all (strong) skin sensitizers<br>☐ No improvements needed<br>☐ Other<br>☐ Comments:
Other, please specify:	__________________________________

Comments: __________________________________

What may improve future risk assessment of skin sensitizers on an EU level, regarding the risk assessment methodology?
(Select all that apply)

- ☐ Harmonization across areas of toxicology
- ☐ Improvements of non-animal test
- ☐ Improvements in the understanding of the underlying mechanisms of skin sensitization
- ☐ More comprehensive exposure data for skin contact to consumer and/or occupational products
- ☐ More data concerning aggregated exposures
- ☐ Data to correct for mixture effects
- ☐ Hazard based assessment
- ☐ Risk assessment based on elicitation levels
- ☐ No improvements needed
- ☐ Other, please specify:
- ☐ Comments:

Other, please specify: __________________________________

Comments: __________________________________

Which method(s) do you use regarding tests/investigations of skin sensitizers?
(Select all that apply)

- ☐ Assessment based on ingredient labelling
- ☐ Chemical analysis of products for skin sensitizers
- ☐ Classification (e.g., GHS/CLP categories 1, 1A, and 1B)
- ☐ Elicitation/patient studies
- ☐ Next Generation Risk Assessments (NGRA) using only New Approach Methods / Non-animal methods
- ☐ Dermal sensitization quantitative risk assessment (QRA) for fragrance ingredients (as described by SCCS 2008) or the revised version QRA2 (as described by SCCS 2017)
- ☐ Other
- ☐ We do not perform risk assessments

Other, please specify: __________________________________

How do you correct for mixture effects in the risk assessment of skin sensitizers in mixtures?

- ◯ We use a correction factor - if possible, please enter the value:
- ◯ We assume additive effects in mixtures of skin sensitizers
- ◯ We do not take the mixture effect into account
- ◯ Other

Please, specify correction factor: __________________________________

Other, please specify: __________________________________

Do you your-self generate data for this purpose? (Select all that apply)	☐ In vivo (human)<br>☐ In vivo (animal)<br>☐ In vitro<br>☐ In chemico<br>☐ In silico<br>☐ A combination of in chemico and in vitro e.g., for use in Defined Approaches as described by OECD guideline No. 497 (2021 OECD Guidelines for the Testing of Chemicals)<br>☐ No
Is this done (Select all that apply):	☐ In-house<br>☐ By external bodies<br>☐ Unknown
May we contact you for further information?	◯ Yes<br>◯ No
Yes selected, please specify:	
Organization:	__________________________________
Contact person:	__________________________________
Email:	__________________________________
Thank you for participating.<br>Feel free to leave any comments:	____
